# Eosinophilic Cystitis: A Rare Cause of Hematuria in Children

**DOI:** 10.1155/2012/710230

**Published:** 2012-11-25

**Authors:** K. S. Venkatesh, Shaila Bhat

**Affiliations:** ^1^Department of Urology, Kasturba Medical College, Manipal University, Karnataka 576104, India; ^2^Department of Pathology, Melaka Manipal Medical College, Manipal University, Karnataka 576104, India

## Abstract

A 7-year-old boy presented with a history of acute onset of hematuria, dysuria, and suprapubic pain. Urine routine and microscopy showed 40–45 red cells/high power field. Urine culture was sterile. Radiological investigations showed a focal mucosal lesion with bladder wall thickening. Biopsy of the lesion revealed an edematous mucosa with florid infiltration by eosinophils into the muscularis propria with focal areas of myonecrosis. He was diagnosed as a case of eosinophilic cystitis. The patient received 6 weeks of tapered prednisone therapy. He was asymptomatic on followup.

## 1. Introduction

Eosinophilic cystitis (EC) is a poorly understood inflammatory condition, often mistaken for a bladder tumor due to its propensity to present with hematuria and bladder wall thickening in children. This entity is rare in the pediatric age group with few cases mentioned in the literature. The condition was initially described in 1960 by Brown [1]. It is characterized by extensive eosinophilic infiltration of all layers of the bladder wall, probably induced by a regulatory disorder of the immune system. This disorder is thought to be due to an antigenic stimulation resulting in IgE-mediated activation of eosinophils causing mast cell degranulation subsequently accounting for the damage to the bladder wall and release of inflammatory mediators [2]. EC presents with a spectrum of urological symptoms as varied as increased frequency, hematuria, and suprapubic pain. Current treatment approaches include observation, identification of incriminating allergens, medical modalities such as steroids, antihistamine, and antibiotics. In cases of intractable hematuria, transurethral bladder resection and partial cystectomy have been employed [2]. This case seeks to highlight the alarming presentation and consequent management of this rare entity.

## 2. Case Presentation

**Figure 1:**
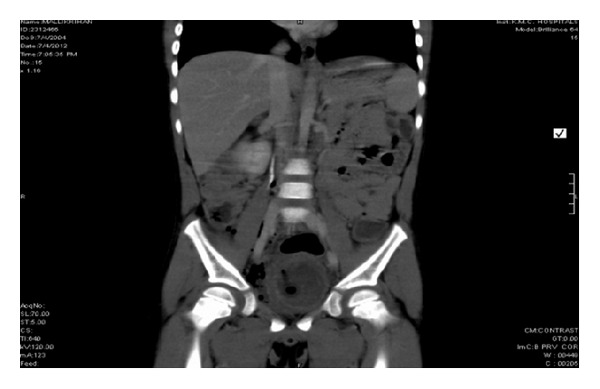
Contrast CT showing diffuse thickening of the right posterolateral bladder wall.

**Figure 2:**
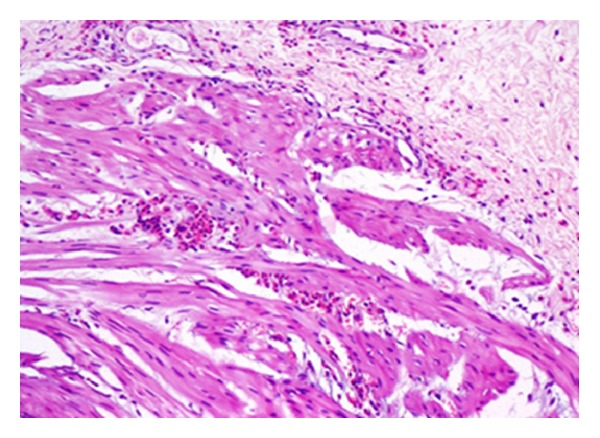
Eosinophilic infiltration into the right posterior muscle bundle (H&E ×400).

 Our patient was a seven-year-old male who presented with a history of acute onset hematuria, dysuria and suprapubic pain. He also gave history of frequency, urgency, urge urinary incontinence and passage of blood clots in urine. There was no antecedent history of fever, upper respiratory infection or flank pain. There was no history of bleeding from any other site or of similar episodes in the past. There was no past history of allergy. The child had an uneventful birth history and had attained normal developmental milestones. On evaluation, he had normal blood pressure, hemoglobin of 12.3 g/dL, total count of 9.4 × 10^3^/*μ*L with 38.9% neutrophils, 45.8% lymphocytes, 8.9% monocytes, 5.7% eosinophils, and 0.7% basophils. Peripheral smear was normocytic normochromic. Urine routine and microscopy showed 40–45 red cells/high power field and 25–30 pus cells/high power field. Urine-specific gravity was 1.030, and urine nitrite was negative. Renal function test was normal. Complement C3 was 109.0 mg/dL. Urine culture was sterile, urine PCR for adenovirus and mycobacterium tuberculosis was negative. Radiological investigations, like USG, showed a focal mucosal lesion with bladder wall thickening in the posterior and right lateral wall of the bladder. Contrast-enhanced CT abdomen showed minimally enhancing smooth eccentric thickening of the wall predominantly on the right side and along the base of the urinary bladder (Figure 1). The patient underwent cystoscopy which revealed a 4 × 3 cm erythematous, polypoidal lesion superolateral to the right ureteric orifice. Considering the above radiological and cystoscopy features, a working diagnosis of rhabdomyosarcoma was made. Transurethral resection biopsy of the lesion was done, and the bladder was catheterized. Biopsy revealed an edematous bladder mucosa, congested blood vessels and infiltration by eosinophils into the muscularis propria with focal areas of myonecrosis (Figure 2). The patient was treated with 6 weeks of tapered prednisone. He was symptom-free when reviewed after two months. 

## 3. Discussion

Eosinophilic cystitis is a rare, poorly understood inflammatory disorder involving the urinary bladder. Following its initial description by Brown in 1960, less than 40 cases of EC have been reported in the pediatric age group [3, 4]. As in the adults, males are more commonly affected in the pediatric population with a mean age of 6 years [5, 6]. Our patient was a 7-year-old male. The exact etiology of EC remains elusive though an allergic response has been proposed. The postulated allergens include food allergens, dust mite, pollen, condom antigens, iodine, and anesthetic ointments. Asthma and celiac disease are also known to be associated with EC. No specific allergen was found in this case. The pathogenesis involves IgE-mediated activation of eosinophils with subsequent mast cell degranulation and muscle damage. The patients present with a spectrum of urological symptoms such as increased frequency, hematuria, suprapubic pain, dysuria, and diurnal and nocturnal enuresis. Cases in the pediatric age group can present with a palpable suprapubic mass [7]. Peripheral blood eosinophilia has been present in many cases but not in the range of the hypereosinophilic syndrome. Our patient had a normal differential count with the eosinophil count within the normal range. A positive urine culture has been reported in a few cases of EC. The urine culture was negative in our patient. Eosinophils are rarely identified in the urinary sediment because they are rapidly degraded or there is little mucosal shedding [8]. Imaging studies in EC patients can closely mimic an infiltrating mass suggestive of a tumor and bladder wall thickening along with hematuria, and the cystoscopy findings as was seen in this patient often result in the suggestion of an initial diagnosis of a rhabdomyosarcoma of the bladder. The intense inflammatory changes which include vascular congestion and edema in the bladder wall associated with this lesion may produce heaped-up excrescences, which resemble vesical rhabdomyosarcoma [4]. It is imperative to obtain adequate deep biopsies in order to study the muscle involvement; or else, the diagnosis can be missed. Due to the rarity of this disorder in children, there are no guidelines for optimal treatment and followup. The treatment is empirical [9]. The first line of management usually comprises the removal of any suspected allergen, followed by antihistamines and corticosteroids. It has been proposed that corticosteroids, with their anti-inflammatory action, may hasten the resolution of symptoms by stabilizing lysosomal membranes [10]. In refractory cases, oral cyclosporine A for 8 months, montelukast sodium for children who present with peripheral eosinophilia, and 6 intravesical DMSO instillation twice a week, 50 mL/50% for 1 hour have been tried. Eosinophilic cystitis in children usually follows a benign, self-limited course, although progression to fibrosis of bladder with secondary obstructive uropathy remains a possibility and has been seen in some of the cases reported. The appropriate diagnosis and management hinges on clinical suspicion coupled with a histopathological evaluation.

## 4. Conclusion

Eosinophilic cystitis in the pediatric age group frequently masquerades as a tumor, causing anxiety. Eosinophilic cystitis, although a rare diagnosis, has to be considered in a child presenting with urinary symptoms coupled with the more sinister bladder wall thickening. A deep biopsy with histopathological evaluation is the key to the diagnosis in order to proceed with the varied treatment options.
